# Development of Chloroplast Genomic Resources in Chinese Yam* (Dioscorea polystachya)*

**DOI:** 10.1155/2018/6293847

**Published:** 2018-03-14

**Authors:** Junling Cao, Dan Jiang, Zhenyu Zhao, Subo Yuan, Yujun Zhang, Teng Zhang, Wenhao Zhong, Qingjun Yuan, Luqi Huang

**Affiliations:** ^1^State Key Laboratory Breeding Base of Dao-di Herbs, National Resource Center for Chinese Materia Medica, China Academy of Chinese Medical Sciences, Beijing 100700, China; ^2^Center for Postdoctoral Research, China Academy of Chinese Medical Sciences, Beijing 100700, China; ^3^Dongzhimen Hospital, Beijing University of Chinese Medicine, Beijing 100700, China; ^4^School of Chinese Materia Medica, Beijing University of Chinese Medicine, Beijing 102488, China; ^5^Department of Immunology, Medical College, Wuhan University of Science and Technology, Wuhan 430065, China

## Abstract

Chinese yam has been used both as a food and in traditional herbal medicine. Developing more effective genetic markers in this species is necessary to assess its genetic diversity and perform cultivar identification. In this study, new chloroplast genomic resources were developed using whole chloroplast genomes from six genotypes originating from different geographical locations. The* Dioscorea polystachya* chloroplast genome is a circular molecule consisting of two single-copy regions separated by a pair of inverted repeats. Comparative analyses of six* D. polystachya* chloroplast genomes revealed 141 single nucleotide polymorphisms (SNPs). Seventy simple sequence repeats (SSRs) were found in the six genotypes, including 24 polymorphic SSRs. Forty-three common indels and five small inversions were detected. Phylogenetic analysis based on the complete chloroplast genome provided the best resolution among the genotypes. Our evaluation of chloroplast genome resources among these genotypes led us to consider the complete chloroplast genome sequence of* D. polystachya *as a source of reliable and valuable molecular markers for revealing biogeographical structure and the extent of genetic variation in wild populations and for identifying different cultivars.

## 1. Introduction

Chinese yam (*Dioscorea polystachya *Turcz.) belongs to section* Enantiophyllum* in genus* Dioscorea*, which also includes economically important food yams of tropical origin such as* D. alata* (water yam) and* D. rotundata* (white guinea yam) [1]. It is allogamous with fleshy tuber, branched stems, papery to thinly leathery leaves, and its seeds are inserted near middle of capsule and winged all round [2]. Chinese yam originated in China and was domesticated in the Song Dynasty, dating back approximately 1000 years [3]. It has been used as a dietary food and as a traditional medicine for strengthening stomach function, alleviating anorexia, and treating diarrhea [4].

Nowadays, there are mainly 80 cultivars on the Chinese market [5]. For a long time, cultivated yams mainly rely on clonally propagated using vegetative propagation of tubers, which led to serious degradation [3]. Its production systems face the problem that the cultivars have the limited diversity during long-term vegetative reproduction [6]. Detailed analysis of the genetic diversity in this species is important, because an accurate assessment of the genetic structure and diversity of cultivated and wild yams can be invaluable in crop breeding for diverse applications [7]. For example, analysis of the genetic variability among cultivated and wild yams can facilitate understanding of the process of domestication followed by Chinese farmers to generate agricultural biodiversity. However, there is lack of adequate information on the diversity evaluation of Chinese yam. Providing the potential conservation approaches for sustainable use, thereby saving the genetic diversity of this species in nature, is important.

Molecular resources have recently been developed in Chinese yam. For example, random-amplified polymorphic DNA (RAPD), inter-simple sequence repeats (ISSR), intron sequence amplified polymorphism (ISAP), and sequence characterized amplified region (SCAR) markers have been used to examine the genetic relationships among different cultivars and identify the most popular cultivar [3, 8–10]. However, these markers have low diversity, stability, and reproducibility. The development of more effective genetic markers will be necessary to assess genetic diversity and identify cultivars.

Recently, the chloroplast genome has been developed with the availability of the next-generation sequencing [11]. The chloroplast genomes of higher plants harbor approximately 130 genes in a 120–160 kb sequence [12]. Chloroplast genomes usually have a circular structure consisting of two copies of the large inverted repeat (IR) region separated by small single-copy (SSC) and large single-copy (LSC) regions and exhibit highly conserved gene content and order [13]. The nucleotide substitution rate of chloroplast genes is lower than that of nuclear genes but higher than that of mitochondrial genes [14, 15]. Most protein-coding genes (83 or 81 genes) have been used for phylogenetic analyses and have proven to be effective in resolving difficult phylogenetic relationships [16–18]. Noncoding regions are most likely to evolve faster than coding regions in the chloroplast genome, and, therefore, these mutation “hot spots” have been used to identify species and clarify relationships at lower taxonomic levels [19–23].

Chloroplast genomes are typically uniparentally inherited, which may greatly facilitate the use of chloroplast genome markers in plant population genetic studies [24]. Chloroplast genome markers, such as single nucleotide polymorphisms (SNPs) and simple sequence repeats (SSRs), have been used to monitor gene flow, population differentiation, and cytoplasmic diversity [25–28]. These chloroplast genome markers can also be applied to investigate domestication processes, such as the evolutionary history of* Scutellaria baicalensis* [29]. Another application of chloroplast genome markers is phylogeographical analysis, because the uniparental inheritance shows a clearer geographical structure than nuclear markers do [30]. The cultivars yam also is clonally propagated. Herein, we sequenced six wild* D. polystachya *genotypes from different geographical locations using the Illumina HiSeq platform. The first objective was to evaluate the intraspecific variation in this species, and the second objective was to obtain useful chloroplast molecular markers, including SNPs, SSRs, and indels, for evolutionary studies by comparing the chloroplast genomes. The genomic and marker resources developed in this study will not only reveal biogeographical structure and extensive population genetic variation in the wild populations of* D. polystachya* but also provide a molecular toolkit for cultivar identification.

## 2. Materials and Methods

### 2.1. Plant Materials, DNA Extraction, and Sequencing

In total, six genotypes of* D. polystachya* were used (Table 1). Chinese yam was obtained from Hebei, Shandong, Henan, Beijing, Jiangsu, and Fujian, China, to represent the geographical distribution of this species. Voucher specimens were deposited in herbaria of the Institute of Chinese Materia Medica (CMMI), China Academy of Chinese Medical Sciences. Fresh leaves of each accession were immediately dried with silica gel prior to DNA extraction. Total genomic DNA was isolated from each individual plant using the mCTAB extraction protocol [31] and purified using the Wizard DNA Cleanup System (Promega, Madison, WI, USA). DNA samples were randomly fragmented into 400–600 bp lengths using an ultrasonicator. An Illumina paired-end DNA library with 500 bp insert size was constructed using a NEBNext® Ultra™ DNA Library Prep Kit following the manufacturer's instructions. Paired-end sequencing (2 × 150 bp) was conducted on an Illumina HiSeq X Ten platform.

### 2.2. Assembly and Annotation

The paired-end reads were qualitatively assessed and assembled using SPAdes 3.6.1 [32]. Chloroplast genome sequence contigs were selected from the initial assembly by performing a BLAST search using the* Dioscorea elephantipes* chloroplast genome sequence as a reference (GenBank accession number: EF380353). The selected contigs were assembled with Sequencher 5.4.5 (http://www.genecodes.com/). Gaps in the contigs were filled by PCR amplification and Sanger sequencing. The four junctions between the IRs and the SSC/LSC regions were checked by amplification with specific primers followed by Sanger sequencing [33]. The chloroplast genome annotation was performed with Plann [34] using the* D. elephantipes *reference sequence from GenBank. The chloroplast genome map was drawn using Genome Vx software [35].

### 2.3. Molecular Marker Development and Validation

All sequenced* D. polystachya* chloroplast genomes were aligned using MAFFT v7 [36], assuming collinear genomes for the full alignment, and then adjusted manually using Se-Al 2.0 [37]. Variable and parsimony-informative base sites across the complete chloroplast genomes were calculated using MEGA 6.0 software [38].

The chloroplast genome sequences were analyzed to identify potential microsatellites (simple sequence repeats) using MISA software (http://pgrc.ipk-gatersleben.de/misa/). The minimum numbers (thresholds) for the SSR motifs were 10, 5, 4, 3, 3, and 3 for mono-, di-, tri-, tetra-, penta-, and hexanucleotide repeats, respectively. All of the repeats found were manually verified, and redundant results were removed.

Based on the aligned sequence matrix, the microstructural events were checked manually and were further divided into three categories: (i) SSR, (ii) non-SSR-related indels (common indels), and (iii) inverted sequences. Using the XSW genotype genome sequence as the standard reference, the size, location, and evolutionary direction of the microstructural events were counted. The proposed secondary structures of the inverted regions were analyzed using mfold software [39].

### 2.4. Phylogenetic Reconstruction

Phylogenetic analysis was conducted using the chloroplast genome sequences of six genotypes of* D. polystachya *and four other* Dioscorea *species with available chloroplast genome sequences from GenBank (*D. nipponica*,* D. villosa*,* D. zingiberensis*, and* D. elephantipes*).* Tacca chantrieri *was used as an outgroup. Sequence alignments were carried out using MAFFT v7 [36] and then adjusted manually using Se-Al 2.0 [37]. We performed independent phylogenetic analyses using Bayesian inference (BI) and maximum likelihood (ML). RAxML version 8.0.20 was used for ML analyses with the GTR + G model. Node support values were determined with 500 rapid bootstrapping replicates. MrBayes 3.2.2 [40] was used to perform a BI analysis. The Markov chain Monte Carlo (MCMC) analysis was run for 2 × 5,000,000 generations. The average standard deviation of split frequencies remained below 0.01 after the fifty percent burn-in. The remaining trees were used to build a 50% majority-rule consensus tree.

## 3. Results

### 3.1. Chloroplast Genome Sequencing, Characterization, and Annotation

Using the Illumina HiSeq X Ten system, the total DNA from six genotypes of* D. polystachya* was sequenced to produce 47,638,574–70,997,840 paired-end raw reads (150 bp average read length) per genotype. After de novo and reference-guided assembly, the finished, high-quality chloroplast genome sequences of these six genotypes of* D. polystachya* were obtained. The chloroplast genome sequences were deposited in GenBank (Table 1).

The* D. polystachya* chloroplast genomes ranged from 153,243 to 153,292 base pairs in length. The chloroplast genome can be divided into four regions: a pair of IR regions, a LSC region, and a SSC region. The overall GC content of the chloroplast genome was 37%, which is consistent with those of previously reported* Dioscorea *species [41]. The GC contents of the LSC and SSC regions were 34.8% and 30.9%, respectively, while that of the IR region was 42.9% (Table 1).

**Figure 1:**
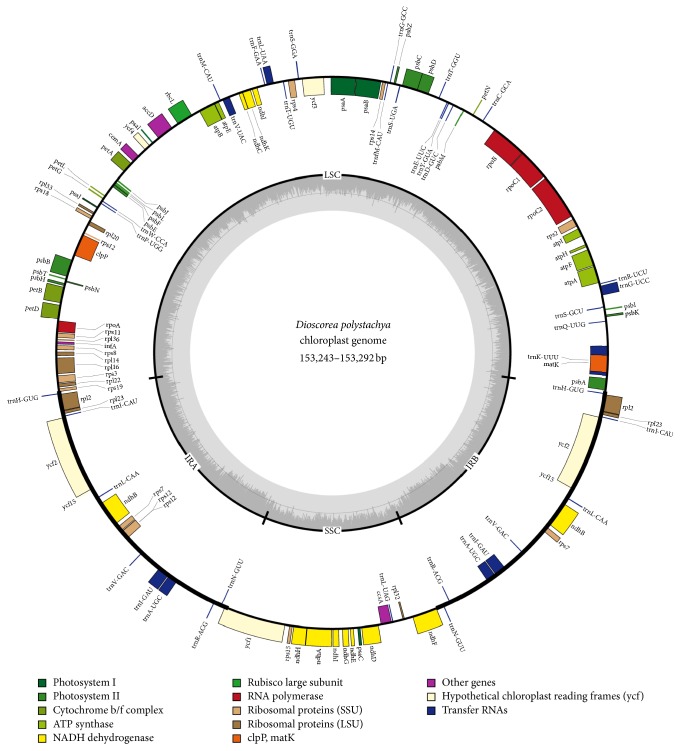
Map of the* Dioscorea polystachya* chloroplast genome. The genes inside and outside of the circle are transcribed in the clockwise and counterclockwise directions, respectively. Genes belonging to different functional groups are shown in different colors. Thick lines indicate the extent of the inverted repeats (IRa and IRb) that separate the small single-copy (SSC) and large single-copy (LSC) regions of the genome.

A total of 112 unique genes were identified in the* D. polystachya* chloroplast genome, including 79 protein-coding genes, 29 tRNA genes, and 4 ribosomal RNA genes (Figure 1 and Table 2). A total of 62 protein-coding and 22 tRNA genes were located in the LSC region, while 12 protein-coding genes and one tRNA gene were located in the SSC region. All the rRNA genes were located in the IR region, along with six protein-coding (*ndhB*,* rpl23*,* rps7*,* rps12*,* ycf2*, and* rpl2*) genes and eight tRNA* (trnA-UGC, trnH-GUG, trnI-CAU, trnI-GAU, trnL-CAA, trnN-GUU, trnR-ACG, and trnV-GAC)* genes.

The* D. polystachya* chloroplast genome contained 18 intron-containing genes. Among them, sixteen genes had a single intron (ten protein-coding and six tRNA genes) and two genes (*clpP* and* ycf3*) contained two introns. The* trnK-UUU* gene had the largest intron, which contained the* matK* gene. The* rps12* gene was trans-spliced, with the 5′ end located in the LSC region and the duplicated 3′ end in the IR region.

### 3.2. Numbers and Pattern of SNP Mutations

The length of the alignment of the six chloroplast genomes was 153,497 bp. In total, 141 SNPs were detected, 84 of which were found in the LSC region, 7 in the IR region, and 43 in the SSC region (Table S1). A total of 134 of these SNPs were found in the IRs, 54 of which were in intergenic spacers, 70 in coding region, and 10 in intron regions. Twenty coding regions harbored SNPs;* ycf1* had the highest number of SNPs (19), followed by* rpoC2* (five), and* rpoB* (five). Five intron regions harbored SNPs (four in* atpF*, two in* trnG* and* rpoC1*, and one in* trnV* and* rps16*).

**Figure 2:**
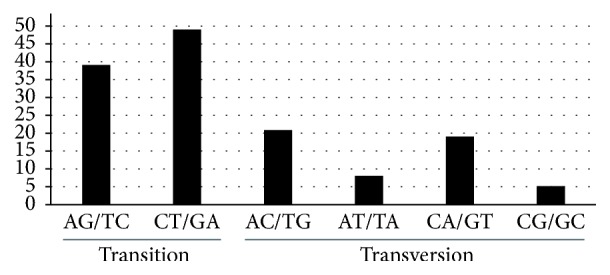
The patterns of nucleotide substitution among the six* D. polystachya* chloroplast genomes. The patterns were divided into six types as indicated by the six non-strand-specific base-substitution types (i.e., numbers of considered G to A and C to T sites for each respective set of associated mutation types).

The pattern of SNP mutations is shown in Figure 2. A total of 88 transitions (Ts) and 53 transversions (Tv) were present, and the Tv to Ts ratio was 1 : 0.6, indicating a bias in favor of transitions. The most frequently occurring SNP mutations were from C to T and from G to A; mutations from C to G and from G to C exhibited the lowest frequency.

### 3.3. Microsatellites

**Figure 3:**
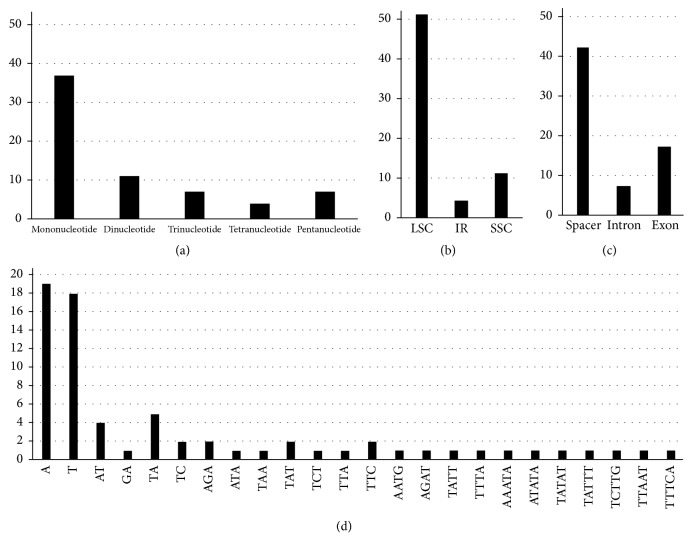
Analyses of simple sequence repeats (SSRs) in the* D. polystachya* chloroplast genomes. (a) Number of different SSR types detected by MISA. (b) Number of SSRs in the LSC, SSC, and IR regions. (c) Number of SSRs in spacers, exons, and introns. (d) Frequency of identified SSR motifs in the different repeat classes.

With MISA analysis, 66 SSR loci were detected in the* D. polystachya* chloroplast genome. These SSRs included 37 mononucleotide motifs, which ranged in length from 10 to 16 nucleotides, and 11 dinucleotide, 7 trinucleotide, 4 tetranucleotide, and 7 pentanucleotide SSRs (Figure 3). Among the 48 mononucleotide and dinucleotide SSRs, 46 contained only A or T. Most SSRs were located in the noncoding portions of the LSC and SSC regions. After in silico comparative analysis, twenty-four SSR loci showed polymorphisms among the six genotypes of* D. polystachya* (Table 3). The* clpP* intron had the highest number of polymorphic SSRs (three), followed by* matK-trnK* and* psbE-petL* with two polymorphic SSRs. The other fifteen spacers and the* rpl16* intron contained only one polymorphic SSR each (Table S2). We designed primer pairs to amplify those SSRs and the other 42 SSR loci (Table S3).

### 3.4. Indels

**Figure 4:**
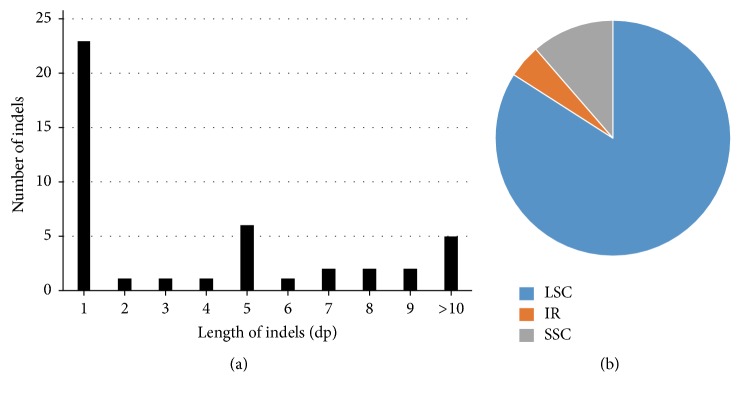
Indels identified in the chloroplast genomes of* D. polystachya.* (a) Numbers of individual indels shown by sequence length. (b) Relative frequency of indel occurrence in the LSC, SSC, and IR regions.

The indels involving SSR polymorphisms were filtered out of these analyses. We retrieved 44 common indels from the* D. polystachya* chloroplast genomes (Table S4). No indels were found in the coding regions. A total of 27 spacer regions harbored indels; the* psbM-trnD* and* rbcL-accD* spacer had the highest number of indels (three), followed by* trnK-trnQ*,* psbI-trnS*,* trnS-trnG*,* petN-psbM*,* trnT-psbD*,* trnF-ndhJ*,* psbE-petL*, and* trnL-rpl32*, all containing two indels. The other spacer regions contained only one indel (Table S4). Five indels were located in intronic regions, including the* atpF *(two indels) and* clpP *(three indels) introns. The sizes of the indels ranged from 1 to 28 bp, with one bp indels being the most common (Figure 4). The largest indel, found in the* atpF* intron with a 28 bp length, was a deletion in the MHW genotype. The second longest, which was found in* rbcL-accD*, was an insertion in the YTW genotype. Finally, 13 insertion and 9 deletion indels were specific to the NJW genotype, 12 insertion and 5 deletion indels to YTW, one insertion in the* psbZ-trnG* region to XSW, and one insertion in* trnL-rpl32* region and one deletion in* atpF* intron to MHW. Two deletions in* petN-psbM* and* psbM-trnD *independently occurred in the YTW and NJW genotypes.

### 3.5. Small Inversions

Five small inversions of 2 to 51 bp were identified in the* D. polystachya* chloroplast genomes. All of the inversions and their flanking inverted repeat sequences could form stem-loop structures. The flanking repeats were from 6 to 22 bp in length (Table 4). Two inversions occurred in the LSC region and three in the SSC region. Inversions in the* trnK-matK* spacer,* ndhA* intron, and* ndhD* occurred in the NJW genotype, while an inversion in* ccsA-trnL* occurred in YTW. An inversion in the* trnL* intron occurred in the YTW and MHW genotypes.

### 3.6. Phylogenetic Analysis

**Figure 5:**
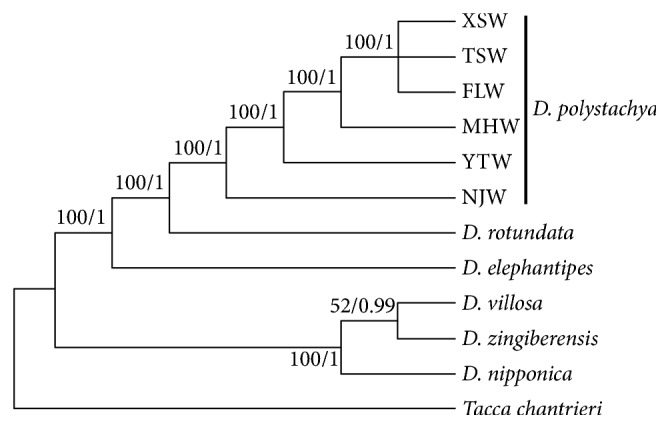
Phylogenetic relationships among* Dioscorea* species constructed from complete chloroplast genome sequences using maximum likelihood (ML) and Bayesian inference (BI). The ML topology is shown, with the ML bootstrap support value/Bayesian posterior probability given for each node.

The phylogenetic position of* D. polystachya *within the genus* Dioscorea *was established using complete chloroplast genomes (Figure 5). The chloroplast genome of* Tacca chantrieri *was used as the outgroup. The ML and BI trees reconstructed were congruent, and both phylogenetic trees had high support. The six* Dioscorea *species were grouped into two branches with 100% bootstrap support, and the NJW genotype was the earliest diverging lineage in* D. polystachya*. The XSW, TSW, and FLW genotypes formed a monophyletic clade.

## 4. Discussion

In this study, we obtained the chloroplast genomes of six* D. polystachya* genotypes using NGS methods, which provided important resources for the discovery of molecular markers. Understanding the genetic relationship of* D. polystachya* is vital to breeding programs and conservation strategies. The* D. polystachya *chloroplast genome exhibited a typical circular structure and was similar in genome size and GC content to the other published* Dioscorea *chloroplast genomes [41]. Using these chloroplast genome data, we were able to develop genetic resources, including SNPs, microsatellites (simple sequence repeats), indels, and small inversions, that constitute essential tools for studies of evolution, population genetics, and the origin of domestication in this species. This information will facilitate the establishment of an effective DNA-barcoding-based identification method and provide valuable markers to study the population genetics of* D. polystachya*.

Among the six genotypes examined, only 141 SNPs were detected. Despite the higher AT content in chloroplast genomes, AT to TA and GC to CG transversions were found to occur significantly less frequently among the four types of transversions (Figure 2). This result clearly indicates a bias in chloroplast genome evolution. In general, most SNPs occurred in the noncoding regions of the* D. polystachya* plastid genomes, which may undergo less natural selection. However, no significant difference was present in the distribution of mutations among the genome regions (Table S1). Variations in mutation rates can be related to the function of genes.* ycf1* had the highest number of SNPs (19) in the* D. polystachya *chloroplast genome, while* atp*,* psa*, and* psb* exhibited the lowest evolutionary rates (Table S1). The* ycf1* gene is the second longest gene; it is essential for plant viability and encodes Tic214, a vital component of the* Arabidopsis *TIC complex [42]. The two parts of* ycf1* in the SSC region (ycf1a and ycf1b) were highly variable in flowering plants [19, 43] and are suitable as markers for phylogeny and species identification [44].

Moreover, indels are another important class of genetic variation. A total of 43 common indels were identified in the* D. polystachya* chloroplast genomes, all in noncoding regions (Table S3). The indel sizes ranged from 1 to 28 bp. According to our results, the mutation rates of these indels were lower than those of nucleotide substitutions. Most indels were specific to individual genotypes, and many were informative for evolutionary studies.* trnL-F*,* rbcL-accD*, and* trnS-trnG* constitute the most frequently applied markers in plant molecular systematics and DNA barcoding [45–47]. As in previous reports, the variable regions* psbM-trnD* and* rbcL-accD* contained the most indels in these* D. polystachya* chloroplast genomes [19]. Adding indels to phylogenetic analyses significantly increases resolution and support compared to simple substitution-based matrices of chloroplast DNA sequences [48].

SSRs, which consist of tandemly repeated motifs of six base pairs (bp) or less, have become widely used as chloroplast genome markers due to their ability to generate highly informative DNA markers. The most common types are mononucleotide repeats, ranging in size from 10 to 15 nucleotides; the occurrence of di-, tri-, tetra-, penta-, and hexanucleotide repeats is less common [28]. After in silico comparative analysis, we identified 24 SSR loci showing polymorphisms, which may allow investigation of spontaneous gene flow among wild and domesticated* D. polystachya *and phylogeographical studies. Because chloroplast genome sequences are highly conserved, chloroplast genome SSRs are transferable across species; thus, these loci can likely be used in studies of other* Dioscorea *species [28].

In this study, we identified SNPs, indels, microsatellites, and small inversions in Chinese yam by comparative analyses of six chloroplast genomes. These resources will allow the identification of commercial cultivars of Chinese yam and the determination of their purity. Furthermore, chloroplast genomic resources are important for further studies of domestication, population genetics, and phylogenetic analysis, possibly in combination with other informative molecular markers from the mitochondrial and/or nuclear genomes.

## Figures and Tables

**Table 1:** Genes identified in the chloroplast genome of *D. polystachya*.

Genotype	FLW	TSW	YTW	XSW	NJW	MHW
Locality	Shijiazhuang, Hebei, China	Tai'an, Shandong, China	Jiaozuo, Henan, China	Xiangshan, Beijing, China	Nanjing, Jiangsu, China	Minhou, Fujian, China
Raw data no.	70,997,840	47,638,574	61,275,836	64,254,664	63,759,008	62,610,816
Mapped read no.	1,076,604	904,074	5,925,916	1,396,336	876,472	1,119,774
Percentage of chloroplast genome reads (%)	1.52%	1.90%	9.67%	2.17%	1.37%	1.79%
Chloroplast genome coverage (X)	1,054	885	5,799	1,367	858	1,096
Accession number in GenBank	MG267375	MG267376	MG267379	MG267377	MG267380	MG267378
Size (bp)	153,255	153,255	153,292	153,257	153,281	153,243
LSC (bp)	83,456	83,456	83,492	83,458	83,484	83,431
SSC (bp)	18,821	18,821	18,816	18,821	18,815	18,834
IRs (bp)	25,489	25,489	25,492	25,489	25,491	25,489

**Table 2:** Details of the complete chloroplast genomes of six *D. polystachya *genotypes.

Category for genes	Group of gene	*Name of gene*
Photosynthesis related genes	Rubisco	*rbcL*
	Photosystem I	*psaA, psaB, psaC, psaI, psaJ*
	Assembly/stability of photosystem I	^*∗*^*ycf3, ycf4*
	Photosystem II	*psbA, psbB, psbC, psbD, psbE, psbF, psbH, psbI, psbJ, psbK, psbL, psbM, psbN, psbT, psbZ*
	ATP synthase	*atpA, atpB, atpE, * ^*∗*^*atpF, atpH, atpI*
	cytochrome b/f complex	*petA, * ^*∗*^*petB, * ^*∗*^*petD, petG, petL, petN*
	Cytochrome c synthesis	*ccsA*
	NADPH dehydrogenase	^*∗*^*ndhA, * ^*∗*^*ndhB, ndhC, ndhD, ndhE, ndhF, ndhG, ndhH, ndhI, ndhJ, ndhK*

Transcription and translation related genes	Transcription	*rpoA, rpoB, * ^*∗*^*rpoC1, rpoC2*
	Ribosomal proteins	*rps2, rps3, rps4, rps7, rps8, rps11, * ^*∗*^*rps12, rps14, rps15, * ^*∗*^*rps16, rps18, rps19,* ^*∗*^*rpl2, rpl14, * ^*∗*^*rpl16, rpl20, rpl22, rpl23, rpl32, rpl33, rpl36*
	Translation initiation factor	*infA*

RNA genes	Ribosomal RNA	*rrn5, rrn4.5, rrn16, rrn23*
	Transfer RNA	^*∗*^*trnA*UGC*, trnCGCA, trnDGUC, trnEUUC, trnFGAA, trnGGCC, * ^*∗*^*trnGUCC, trnHGUG, trnICAU, * ^*∗*^*trnIGAU,* ^*∗*^*trnKUUU, trnLCAA, * ^*∗*^*trnLUAA, trnLUAG, trnfMCAUI, trnMCAU, trnNGUU, trnPUGG, trnQUUG, trnRACG, trnRUCU, trnSGCU, trnSGGA, trnSUGA, trnTGGU, trnTUGU, trnVGAC, * ^*∗*^*trnVUAC, trnWCCA, trnYGUA*

Other genes	RNA processing	*matK*
	Carbon metabolism	*cemA*
	Fatty acid synthesis	*accD*
	Proteolysis	^*∗*^*clpP*

Genes of unknown function	Conserved reading frames	*ycf1, ycf2*

Intron-containing genes are marked by asterisks (*∗*).

**Table 3:** SSRs identified from in silico comparative analysis of the chloroplast genomes of six *D. polystachya *genotypes.

Position	Region	Location	SSR type	Forward sequence	Reverse sequence	PRODUCT1 size (bp)
*matK-trnK*	LSC	space	(TATAT)3	CCGAGGACAAGGAATCCAATCA	AGGTTCTCCTGAGAGTGAACCA	270
*matK-trnK*	LSC	space	(A)10	CCGAATTGGGCCATAAGACTCT	ACCATGACTGATCCTGAAAGGT	223
*atpA-atpF*	LSC	space	(A)12	TGCCATCACTTCATCAAGACCA	CCTCGGAGCCATGGAAGAAATA	253
*atpH-atpI*	LSC	space	(A)10	TACAGCCAATCCAGCAGCAATA	TGAGTTACTTCTCCACCCGATG	161
*rps2-rpoC2*	LSC	space	(A)15	ACCAAATCAATGATCGGACCAA	TAGTGCACCGTTCAAGACAAGA	255
*rpoB-trnC*	LSC	space	(A)11	AGACAGAATAATTGGGGGTAGGA	ACCCCATCTATGTTTAGGTTGCT	273
*petN-psbM*	LSC	space	(T)12	TGGACCAGTTCTTAACAGAATAATG	GGACATATGGCCGTCGAAAGAA	138
*trnE-trnT*	LSC	space	(T)11	CGATGTCGGATTGGTACACGTA	GCATATGCACTCATTCAAGGACA	183
*trnS-psbZ*	LSC	space	(TA)6	TTCAAGACCGGAGCTATCAACC	GCATGTGGTCGAGGAGAGTTTA	232
*trnF-ndhJ*	LSC	space	(TA)7	GCTCCCTCTTTCTCCTTTGTTC	TACCGCGCACATCACTTAGAT	280
*petA-psbJ*	LSC	space	(A)12	CTTGGCATCTGTGATTTTGGCA	TGTTCCTTTCATTTATCCCGTCA	221
*psbE-petL*	LSC	space	(A)11	CCAAGCTTTACTGTACCGAATCC	TGTGTGTGTCGTGTAGCTTGAT	215
*psbE-petL*	LSC	space	(A)10	ATCAAGCTACACGACACACACA	AGCAGCCAACAGAAAACCAAAA	199
*clpP*	LSC	intron 1	(T)10	CACCCTTCCTTTCGTTGGAGTA	ATCGGGAGTACATTTCAGCGTC	213
*clpP*	LSC	intron 2	(T)11	CACCTTTGGATGCATACGGTTC	TATAGTATAGGGCGGGGTCCAA	163
*clpP*	LSC	intron 2	(T)12	CCGGGTAAAGATCTGTCCGAAT	AGCGTGAAGTGCAATTAGATCA	276
*rps11-rpl36*	LSC	space	(T)12	ACCAATACGTCCATTCCTACGG	TAGGCGTGGACGAATTATGGTG	238
*rps8-rpl14*	LSC	space	(T)10	TCCCTACCCATGACGAACTAGA	ACTCGAGTTTTTGGTGCGATTC	259
*rpl16*	LSC	intron	(T)10	GCTCCTCGCGAATGAAATGATT	GCTCGCGAAACCCTTGTTTATT	275
*rpl16-rps3*	LSC	space	(T)12	CGAGTCACACACTAAGCATAGC	GTTCCCCTACAAACAATTCGCG	279
*rps12-trnV*	IR	space	(ATA)4g(TAA)6	TGGTTCTGCTTCCCCTCTTTTT	GCAAAGGGTCGAGAAACTCAAC	274
*ycf1-rps15*	SSC	space	(T)16	CCATTCAACTGGATCTAGGAGGA	TGTGGATTTTACCGATCGGGAA	241
*rpl32-ndhF*	SSC	space	(T)10	TATCTATACTTATTGCACCAATA	ACCAAGTATTAACCAGTGTTAA	176

**Table 4:** The locations, directions, and lengths of five small inversions.

Location	Region	Length of inversions in cpg (bp)		Direction of the small inversions					
		Length of inversion	Length of inverted repeat	FLW	TSW	YTW	XSW	NJW	MHW
*trnK-matK*	LSC	51	13	-	-	-	-	Inverted	-
*trnL* intron	LSC	4	22	-	-	Inverted	-	-	Inverted
*ndhA* intron	SSC	2	14	-	-	-	-	Inverted	-
*ndhD*	SSC	2	6	-	-	-	-	Inverted	-
*ccsA-trnL*	SSC	3	20	-	-	Inverted	-	-	-

## Supplementary Materials

Supplementary Materials Table S1: the patterns of single nucleotide substitutions among the chloroplast genomes of six* D. polystachya* genotypes. Table S2: polymorphic SSR loci identified in the analyzed material. Positions, locations, types, and polymorphisms are shown. Table S3: primers for the other 42 SSR loci identified in the analyzed material. Table S4: polymorphic indels identified in the analyzed material. Indel events are reported for each of the six* D. polystachya* genotypes.

## References

[1] Hsu K. M. (2013). Molecular phylogeny of dioscorea (Dioscoreaceae) in east and southeast asia. *Blumea*.

[2] Flora of China Editorial Committee (2000). *Flora of China*.

[3] Peng B., Zhang Y., Sun X., Li M., Xue J., Hang Y. (2017). Genetic relationship and identification of Dioscorea polystachya cultivars accessed by ISAP and SCAR markers. *Archives of Biological Sciences*.

[4] BABIL P. (2013). Intra-specific ploidy variations in cultivated Chinese yam (Dioscorea polystachya Turcz.). *Tropical Agriculture and Development*.

[5] Zhuang H., Ni Y., Kokot S. (2015). A comparison of near-and mid-infrared spectroscopic methods for the analysis of several nutritionally important chemical substances in the chinese yam (dioscorea opposita): Total sugar, polysaccharides, and flavonoids. *Applied Spectroscopy*.

[6] Li M., Li J., Liu W. (2014). A protocol for in vitro production of microtubers in Chinese yam (*Dioscorea opposita*). *Bioscience, Biotechnology, and Biochemistry*.

[7] Mohammadi S. A., Prasanna B. M. (2003). Analysis of genetic diversity in crop plants—salient statistical tools and considerations. *Crop Science*.

[8] Zhou Y., Zhou C., Yao H., Liu Y., Tu R. (2008). Application of ISSR markers in detection of genetic variation among Chinese yam (Dioscorea opposita Thunb) cultivars. *Life Science Journal*.

[9] Hua S., Tu Q., Lei F. (2009). Genetic diversity of Dioscorea polystachya Turcz. revealed by RAPD markers. *Journal of Plant Genetic Resources*.

[10] Wu Z. G., Li X. X., Lin X. C. (2014). Genetic diversity analysis of yams (Dioscorea spp.) cultivated in China using ISSR and SRAP markers. *Genetic Resources and Crop Evolution*.

[11] Daniell H. (2016). Chloroplast genomes: diversity, evolution, and applications in genetic engineering. *Genome Biology*.

[12] Sanitá Lima M., Woods L. C., Cartwright M. W., Smith D. R. (2016). The (in)complete organelle genome: exploring the use and nonuse of available technologies for characterizing mitochondrial and plastid chromosomes. *Molecular Ecology Resources*.

[13] Jansen R. K. (2005). Methods for obtaining and analyzing whole chloroplast genome sequences. *Methods in Enzymology*.

[14] Dong W., Xu C., Cheng T., Zhou S. (2013). Complete chloroplast genome of *Sedum sarmentosum* and chloroplast genome evolution in Saxifragales. *PLoS ONE*.

[15] Smith D. R. (2015). Mutation rates in plastid genomes: They are lower than you might think. *Genome Biology and Evolution*.

[16] Xue J.-H., Dong W.-P., Cheng T., Zhou S.-L. (2012). Nelumbonaceae: Systematic position and species diversification revealed by the complete chloroplast genome. *Journal of Systematics and Evolution*.

[17] Jansen R. K., Cai Z., Raubeson L. A. (2007). Analysis of 81 genes from 64 plastid genomes resolves relationships in angiosperms and identifies genome-scale evolutionary patterns. *Proceedings of the National Acadamy of Sciences of the United States of America*.

[18] Moore M. J., Soltis P. S., Bell C. D., Burleigh J. G., Soltis D. E. (2010). Phylogenetic analysis of 83 plastid genes further resolves the early diversification of eudicots. *Proceedings of the National Acadamy of Sciences of the United States of America*.

[19] Dong W., Liu J., Yu J., Wang L., Zhou S., Moustafa A. (2012). Highly Variable Chloroplast Markers for Evaluating Plant Phylogeny at Low Taxonomic Levels and for DNA Barcoding. *PLoS ONE*.

[20] Xu C., Dong W., Li W. (2017). Comparative analysis of six lagerstroemia complete chloroplast genomes. *Frontiers in Plant Science*.

[21] Song Y., Chen Y., Lv J. (2017). Development of Chloroplast Genomic Resources for Oryza Species Discrimination. *Frontiers in Plant Science*.

[22] Shaw J., Lickey E. B., Beck J. T. (2005). The tortoise and the hare II: Relative utility of 21 noncoding chloroplast DNA sequences for phylogenetic analysis. *American Journal of Botany*.

[23] Jiang D., Zhao Z., Zhang T. (2017). The chloroplast genome sequence of Scutellaria baicalensis provides insight into intraspecific and interspecific chloroplast genome diversity in Scutellaria. *Gene*.

[24] Rogalski M. (2015). Plastid genomics in horticultural species: importance and applications for plant population genetics, evolution, and biotechnology. *Front Plant Sci*.

[25] Zhang Y., Iaffaldano B. J., Zhuang X., Cardina J., Cornish K. (2017). Chloroplast genome resources and molecular markers differentiate rubber dandelion species from weedy relatives. *BMC Plant Biology*.

[26] Schroeder H., Hoeltken A. M., Fladung M. (2011). Differentiation of Populus species using chloroplast single nucleotide polymorphism (SNP) markers - essential for comprehensible and reliable poplar breeding. *Plant Biology*.

[27] Diekmann K., Hodkinson T. R., Barth S. (2012). New chloroplast microsatellite markers suitable for assessing genetic diversity of Lolium perenne and other related grass species. *Annals of Botany*.

[28] Ebert D., Peakall R. (2009). Chloroplast simple sequence repeats (cpSSRs): Technical resources and recommendations for expanding cpSSR discovery and applications to a wide array of plant species. *Molecular Ecology Resources*.

[29] Yuan Q.-J., Zhang Z.-Y., Hu J., Guo L.-P., Shao A.-J., Huang L.-Q. (2010). Impacts of recent cultivation on genetic diversity pattern of a medicinal plant, Scutellaria baicalensis (Lamiaceae). *BMC Genetics*.

[30] Perdereau A., Klaas M., Barth S., Hodkinson T. R. (2017). Plastid genome sequencing reveals biogeographical structure and extensive population genetic variation in wild populations of Phalaris arundinacea L. in north-western Europe. *GCB Bioenergy*.

[31] Li J. (2013). A modified CTAB protocol for plant DNA extraction. *Chinese Bulletin of Botany*.

[32] Bankevich A. (2012). SPAdes: a new genome assembly algorithm and its applications to single-cell sequencing. *Journal of Computational Biology*.

[33] Dong W., Xu C., Cheng T., Lin K., Zhou S. (2013). Sequencing angiosperm plastid genomes made easy: A complete set of universal primers and a case study on the phylogeny of saxifragales. *Genome Biology and Evolution*.

[34] Huang D. I., Cronk Q. C. B. (2015). Plann: A command-line application for annotating plastome sequences. *Applications in Plant Sciences*.

[35] Conant G. C., Wolfe K. H. (2008). GenomeVx: Simple web-based creation of editable circular chromosome maps. *Bioinformatics*.

[36] Katoh K., Standley D. M. (2013). MAFFT multiple sequence alignment software version 7: improvements in performance and usability. *Molecular Biology and Evolution*.

[37] Rambaut A. Se-Al: sequence alignment editor. version 2.0. http://tree.bio.ed.ac.uk/software/seal/.

[38] Tamura K., Stecher G., Peterson D., Filipski A., Kumar S. (2013). MEGA6: Molecular Evolutionary Genetics Analysis version 6.0. *Molecular Biology and Evolution*.

[39] Zuker M. (2003). Mfold web server for nucleic acid folding and hybridization prediction. *Nucleic Acids Research*.

[40] Ronquist F., Teslenko M., van der Mark P. (2012). Mrbayes 3.2: efficient bayesian phylogenetic inference and model choice across a large model space. *Systematic Biology*.

[41] Hansen D. R., Dastidar S. G., Cai Z. (2007). Phylogenetic and evolutionary implications of complete chloroplast genome sequences of four early-diverging angiosperms: Buxus (Buxaceae), Chloranthus (Chloranthaceae), Dioscorea (Dioscoreaceae), and Illicium (Schisandraceae). *Molecular Phylogenetics and Evolution*.

[42] Kikuchi S., Bédard J., Hirano M. (2013). Uncovering the protein translocon at the chloroplast inner envelope membrane. *Science*.

[43] Dong W., Xu C., Li C. (2015). ycf1, the most promising plastid DNA barcode of land plants. *Scientific Reports*.

[44] Song Y., Wang S., Ding Y. (2017). Chloroplast Genomic Resource of Paris for Species Discrimination. *Scientific Reports*.

[45] Bakker F. T., Culham A., Gomez-Martinez R. (2000). Patterns of nucleotide substitution in angiosperm cpDNA trnL (UAA)-trnF (GAA) regions. *Molecular Biology and Evolution*.

[46] Taberlet P., Coissac E., Pompanon F. (2007). Power and limitations of the chloroplast trnL (UAA) intron for plant DNA barcoding. *Nucleic Acids Research*.

[47] Gao X., Zhu Y.-P., Wu B.-C., Zhao Y.-M., Chen J.-Q., Hang Y.-Y. (2008). Phylogeny of Dioscorea sect. Stenophora based on chloroplast matK, rbcL and trnL-F sequences. *Journal of Systematics and Evolution*.

[48] Liu J., Provan J., Gao L.-M., Li D.-Z. (2012). Sampling strategy and potential utility of indels for DNA barcoding of closely related plant species: A case study in Taxus. *International Journal of Molecular Sciences*.

